# The British Laryngological and Rhinological Association

**Published:** 1888-08

**Authors:** 


### Society Meetings and Notes.

THE British Laryngological and Rhinological Association held its first annual meeting on June 29th. Sir Morell Mackenzie, Bart., was elected president.

